# AI-driven perioperative risk stratification and complication management in craniomaxillofacial surgery: current progress and future directions

**DOI:** 10.3389/fsurg.2026.1859732

**Published:** 2026-07-06

**Authors:** HaiLian Chen, Shuang Zou, Linlin Zheng

**Affiliations:** Department of Nursing, The Second Affiliated Hospital of Zhejiang University School of Medicine, Hangzhou, China

**Keywords:** artificial intelligence, complication prediction, craniomaxillofacial surgery, perioperative management, risk stratification

## Abstract

Craniomaxillofacial surgery is characterized by complex anatomy, high surgical risks, and diverse perioperative complications. Conventional perioperative management relies heavily on surgeons’ subjective experience and lacks standardized, quantifiable risk stratification tools. Artificial intelligence provides a promising strategy for precise and intelligent perioperative care in craniomaxillofacial surgery. This review summarizes recent advances in AI applications for perioperative risk stratification and complication management, focusing on three core domains: preoperative risk assessment and optimization, intraoperative decision support and safety monitoring, and postoperative risk stratification and outcome quantification. Key challenges are discussed regarding data quality and generalizability, model interpretability and clinical trust, and clinical translation and implementation. Future directions are proposed for multimodal AI, explainable AI, and generative AI to establish personalized perioperative management systems. This review aims to provide a structured reference for the intelligent development of perioperative care in craniomaxillofacial surgery.

## 1 Introduction

Craniomaxillofacial (CMF) surgery covers a wide range of procedures, including craniosynostosis repair, orthognathic surgery, cranioplasty, cleft lip and palate correction, and facial trauma reconstruction (1). These procedures share critical features: intricate surgical anatomy, proximity to vital neurovascular structures, long operative times, and high bleeding risk (2). The quality of perioperative management directly affects patients’ intraoperative safety and long-term functional and aesthetic outcomes. CMF surgery has long been challenged by high anatomical variability and subjective evaluation, with clinical decisions largely dependent on expert experience (3). Establishing an objective, accurate, and personalized perioperative risk stratification system is essential to improve clinical quality in this field.

The rapid advancement of artificial intelligence (AI) has profoundly transformed surgical practice. From machine learning (ML) and deep learning (DL) to generative AI, intelligent systems—including large language models (LLMs)—have shown broad potential in diagnostic assistance and intelligent decision-making (4). In CMF surgery, the clinical value of AI has evolved from auxiliary tools to integrated full-process intelligence. AI not only optimizes conventional applications such as three-dimensional(3D) printing (5) but also enhances diagnostic accuracy, improves risk stratification, enables personalized surgical planning, and standardizes postoperative outcome assessment (6–8). Its application has expanded beyond technical optimization to core components of clinical care.

**Figure 1:**
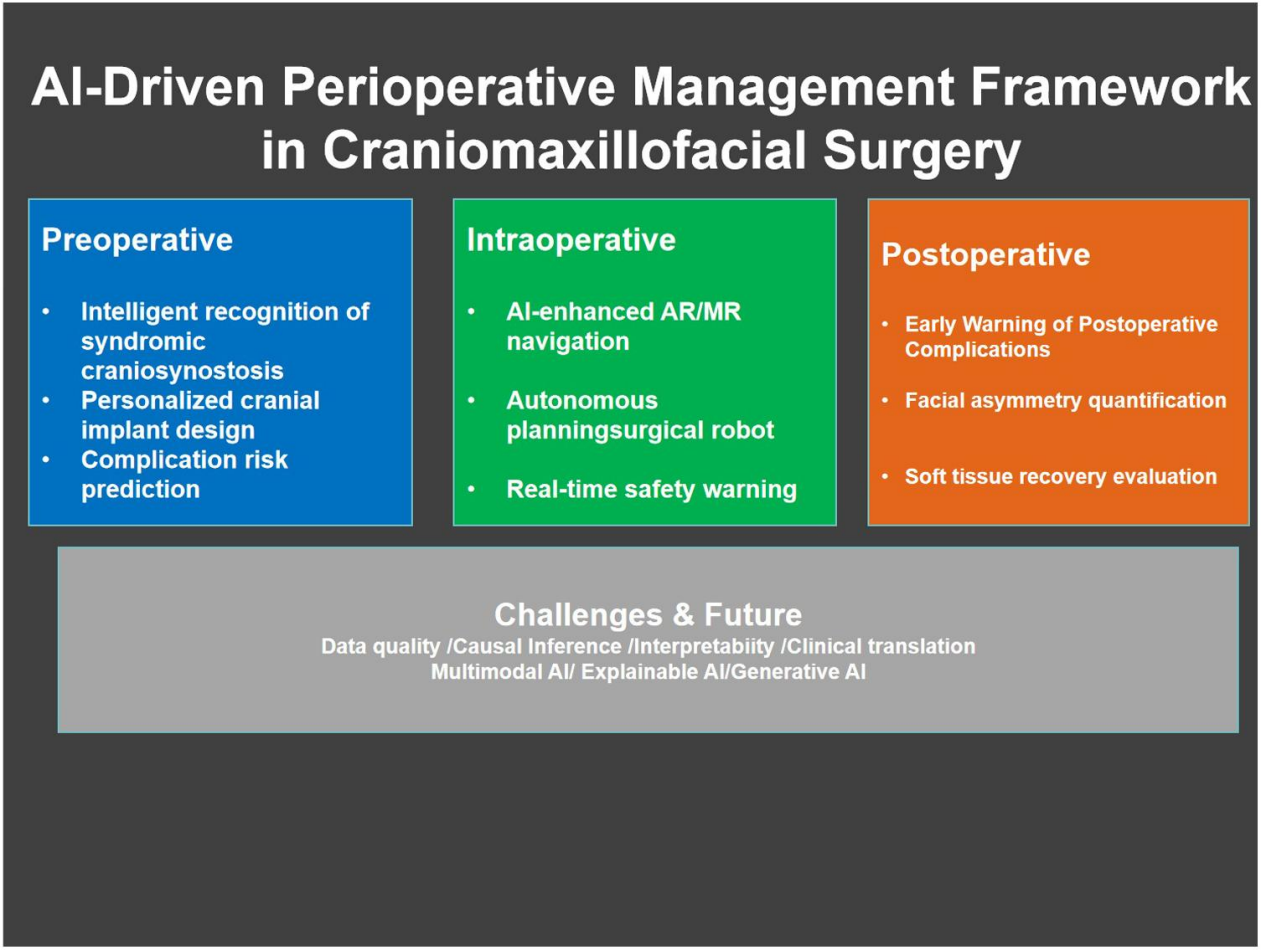
AI-Driven perioperative management framework in craniomaxillofacial surgery.

In recent years, AI-driven perioperative risk stratification and complication management have been increasingly reported across preoperative, intraoperative, and postoperative phases. Nevertheless, current research still faces limitations in model interpretability, external validation, multi-center collaboration, and clinical integration (9). This review summarizes the latest progress of AI in perioperative risk stratification and complication management in CMF surgery, analyzes core challenges, and discusses future directions, aiming to support clinical practice and further research (Figure 1).

## 2 Methods

### 2.1 Search strategy

**Figure 2:**
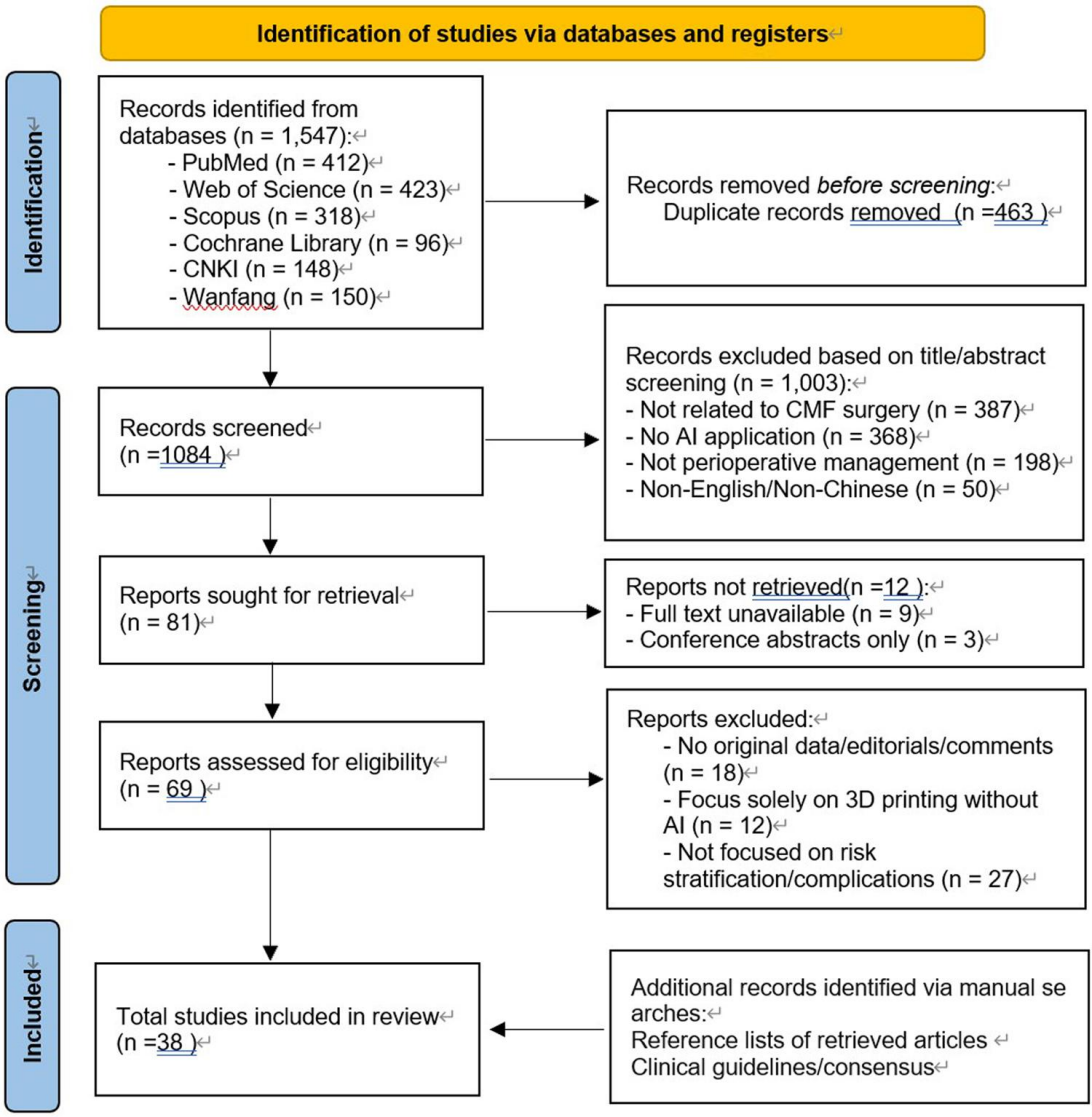
PRISMA 2020 flow diagram of study identification and selection.

This review was conducted in accordance with the Preferred Reporting Items for Systematic Reviews and Meta-Analyses (PRISMA) guidelines to ensure transparency and reproducibility (10). A systematic literature search was performed across the following electronic databases: PubMed, Web of Science, Cochrane Library, CNKI, Wangfang and Scopus, covering the period from January 2000 to March 2026. The search strategy combined keywords and Medical Subject Headings (MeSH) terms related to three domains: (1) artificial intelligence (e.g., “artificial intelligence,” “machine learning,” “deep learning,” “neural network,” “AI”), (2) craniomaxillofacial surgery (e.g., “craniomaxillofacial surgery,” “orthognathic surgery,” “cranioplasty,” “craniosynostosis,” “facial trauma”), and (3) perioperative management (e.g., “perioperative,” “preoperative,” “intraoperative,” “postoperative,” “risk stratification,” “complication prediction”). Additionally, the reference lists of included studies and relevant review articles were manually screened to identify any additional eligible studies. The detailed screening process is presented in the PRISMA flow diagram (Figure 2).

### 2.2 Inclusion and exclusion criteria

Studies were included if they met the following criteria: (1) original research articles applying AI or machine learning techniques to perioperative risk stratification or complication management in craniomaxillofacial surgery; (2) peer-reviewed articles published in English or Chinese; and (3) full text available for data extraction. Exclusion criteria were: (1) conference abstracts, editorials, commentaries, or opinion pieces without original data; (2) studies focusing solely on 3D printing or CAD/CAM without AI components; (3) duplicate publications; (4) studies with insufficient data for extraction.

### 2.3 Study selection and data extraction

Two reviewers (H.L.C. and S.Z.) independently screened the titles and abstracts of all retrieved records against the inclusion criteria. Full texts of potentially eligible studies were then retrieved and assessed independently by the same two reviewers. Disagreements were resolved through discussion or by consultation with a third reviewer (L.L.Z.). A standardized data extraction form was used to collect the following information from each included study: first author, study design, sample size, AI method, validation type, performance metrics, and clinical application status. Extracted data were cross-checked for accuracy. The extracted information was subsequently organized into a structured summary table (Supplementary Table 1).

## 3 Application of AI in preoperative management

### 3.1 Intelligent early recognition of syndromic craniosynostosis

In patients with syndromic craniosynostosis, premature cranial suture fusion causes skull malformation, which further results in intracranial hypertension and neurodevelopmental delay. Affected individuals frequently manifest severe craniofacial abnormalities as well as limb deformities, typical of Crouzon syndrome, Apert syndrome, Pfeiffer syndrome and other related disorders (11). These multisystem malformations not only elevate anesthetic risks and surgical complexity, but also may trigger serious complications including intellectual disability, visual loss and even fatal asphyxiation (12). Accordingly, early identification of syndromic craniosynostosis is critically important for formulating individualized treatment protocols, as well as protecting neurological function and saving lives. Traditional approaches depend on clinical signs and genetic testing. The introduction of AI has markedly improved the efficiency and accuracy of syndrome recognition. Hennocq et al. (13) used deep learning and geometric morphometrics to analyze frontal, lateral, and ear photographs of patients with Apert, Crouzon, Pfeiffer, Muenke, and Saethre-Chotzen syndromes. After extracting geometric and texture features, an extreme gradient boosting (XGBoost) classifier was used for multi-class diagnosis. The model achieved an overall diagnostic accuracy of 70.2% [95% confidence interval (CI): 0.593–0.797] in an independent international validation set, with a balanced accuracy of 84.4% and an area under the curve (AUC) of 0.941 for the Crouzon-Pfeiffer group. The study also revealed milder facial phenotypes in patients with Crouzon-Pfeiffer syndrome carrying fibroblast growth factor receptor 2 (FGFR2) splice donor site variants, with significant phenotypic differences across distinct genotypes. Based on this work, the same research team further validated the real-world applicability of this AI tool in a humanitarian context (14). The researchers applied their algorithm to a publicly available, low-resolution news photograph of a refugee family taken during an armed conflict. The algorithm successfully identified a child with typical craniofacial features of Apert syndrome. This case study suggests that in crisis conditions, AI-based facial recognition technology can be used to screen publicly available media images to identify at-risk patients who might otherwise remain undiagnosed and without access to treatment (14).

### 3.2 Personalized cranial repair and intelligent implant design

Preoperative planning in CMF surgery has traditionally relied on computer-aided design (CAD). Manual implant or bone scaffold design is time-consuming, experience-dependent, and poorly personalized. AI algorithms enable high-precision automatic segmentation of bone structures from CT/MRI images and intelligent inference and reconstruction of defective regions. The SCAI-Net framework (6) uses a V-Net architecture for automatic defect reconstruction from CT images in cranial defect repair. Its direct defect reconstruction pathway completes automatic defect repair within 0.1 s, reducing conventional CAD time from 47 min to 93.5 s, while ensuring mechanical safety and anatomical fit through finite element analysis and marginal gap verification. The framework also uses multi-stage data augmentation and a dual-pathway design, offering flexible options for diverse clinical scenarios. Meticuly's AI system rapidly renders CT data into high-fidelity 3D models (15). The point cloud diffusion model developed by the University of Basel can directly infer the complete healthy skull shape from defective skull CT scans and automatically generate implants, with the entire workflow taking approximately 20 min (16). Zubizarreta-Oteiza et al. (17) applied generative design algorithms to reduce cranial implant mold design time from 2 h to 1.3 min. Combined with stereolithography 3D printing, high-precision resin molds were fabricated with a deviation of only 0.0763 mm after sterilization. The cast polymethyl methacrylate (PMMA) implants showed excellent fitting accuracy, providing a feasible solution for rapid, low-cost, bedside production of personalized cranial implants.

### 3.3 Complication risk prediction

CMF surgery carries a high risk of postoperative complications due to complex anatomy and adjacent vital neurovascular structures, which significantly affect functional recovery and quality of life (18). AI allows preoperative risk prediction of perioperative adverse events and guides preoperative interventions. Stehrer et al. (19) used a random forest algorithm to predict perioperative blood loss in 950 orthognathic patients based on preoperative clinical and laboratory data. Predictions were strongly correlated with actual blood loss (*p* < 0.001), with a mean deviation of 7.4 mL [standard deviation (SD) 172.3 mL]. Bimaxillary surgery, preoperative hematocrit, and hemoglobin were the top three predictive factors. This model enables preoperative identification of high-bleeding-risk patients to support transfusion planning and surgical strategy. Li et al. (7) adopted double machine learning (DML) and the T-learner framework to develope prediction models using a multi-center cohort of 1,368 cranioplasty patients. Nine core predictors were screened from 26 variables. Based on these predictors, the model yielded an AUROC of 0.930 to 0.949 for overall in-hospital complications. Causal inference confirmed subcutaneous negative-pressure drainage [Average Treatment Effect (ATE) = −0.241] and titanium mesh (ATE = −0.191) as modifiable protective factors. Patients were stratified into high and low-risk groups using the Youden index for targeted interventions. Specific models were also established for seven complication subtypes, with optimal architectures selected by discriminative ability, calibration, and clinical utility.

## 4 Application of AI in intraoperative surgery

### 4.1 AI-Driven augmented and mixed reality navigation

Precise intraoperative navigation has always been a challenge in CMF surgery. In the past, surgical procedures relying solely on the surgeon's experience and two-dimensional (2D) images carried significant risks of injury. The integration of AI-driven augmented reality (AR) and mixed reality (MR) technologies enables 3D reconstruction and automatic segmentation of patients’ preoperative CT or MRI data via AI algorithms, generating digital 3D models. Surgeons can directly superimpose the model holographically onto the patient's surgical field at a 1:1 ratio using head-mounted see-through display devices (20). Xie et al. (21) calculates markers in CT scans preoperatively by combining the singular value decomposition (SVD) algorithm (patients wear a personalized custom-made occlusal splint for preoperative CT scanning, with steel balls and ArUco visual markers fixed on the splint), achieving accurate alignment between the virtual model and the patient's anatomy. Intraoperatively, an Intel Realsense L515 red-green-blue depth (RGB-D) camera captures visual markers on surgical instruments for real-time tracking, and Kalman filtering is introduced for post-processing of tracking data to enhance tracking stability. The system ultimately presents navigation information through the HoloLens 2 head-mounted display (HMD), with an average tracking error of 1.55 ± 0.25 mm and a system delay of 75–100 ms. The platform also provides active safety protection for surgeons by delineating high-risk areas preoperatively and calculating the real-time distance between the tip of the surgical instrument and these areas intraoperatively, which is displayed intuitively in the HMD using green, yellow, and red color coding. This integrated approach combining intelligent algorithms with intraoperative navigation technology is gradually transitioning from laboratory research to clinical commercialization. In March 2026 (22), Medtronic's Stealth AXiS system received FDA approval for use in craniocerebral and otolaryngologic surgeries. The system integrates intelligent preoperative planning, high-precision optical navigation, and robotic operation capabilities on a single platform, forming a comprehensive integrated intraoperative navigation solution.

### 4.2 Autonomous planning surgical robots

With the deep integration of AR, AI, and robotics, CMF surgery robots are transitioning from assisted execution to autonomous planning and have gained preliminary autonomous decision-making capabilities through AI and path-planning algorithms. Lin et al. (23) developed the CPSR-I (cranio-maxillofacial plastic surgery robot - I) system and applied it to genioplasty. This system employs an adaptive fuzzy control algorithm and Kalman filtering technology, achieving automatic bone drilling control through force perception and position feedback. Guo et al. (24) reported the application of an autonomous dental implant robot for post-maxillary reconstruction rehabilitation. This system performs intelligent surgical planning based on preoperative CBCT images, automatically determining the position, angle, and depth of the implants. Intraoperatively, it uses an infrared vision system to track the patient's subtle head movements in real time, controlling the robotic arm to autonomously complete implant site preparation and implant placement, while adaptively adjusting drilling parameters using force perception and servo feedback. The system can autonomously move in and out of the patient's oral cavity, achieving precise implantation of multiple implants and immediate restoration under conditions of limited bone volume. An *in vitro* study on zygomatic implant placement compared two robotic techniques (25): fully autonomous planning and a human-robot collaborative hybrid model. The control group used conventional one-step autonomous drilling, while the experimental group used a two-stage hybrid technique: autonomous drilling in the alveolar bone region, followed by passive mode in which the surgeon guided the robotic arm to complete zygomatic site preparation under robotic axial guidance. Although the hybrid technique showed slightly lower coronal accuracy (entry and apex deviation ∼1 mm, angle <5°), total drilling time was significantly reduced from 605.27 s to 206.07 s (*P* < 0.001). Autonomous planning surgical robots, through the integration of AI and force control, have not only improved surgical accuracy and efficiency but also provided reliable evidence for the clinical application of human–machine collaboration in complex CMF surgery.

## 5 Application of AI in postoperative management

### 5.1 Early warning of postoperative complications

Early postoperative complications act as dynamic warning indicators, enabling early identification of high-risk patients, enhanced surveillance, and prompt intervention to prevent severe adverse events. Matar et al. (26) developed machine learning models for osteoradionecrosis (ORN) risk stratification after fibula free flap reconstruction in oral cancer patients, with the stacked ensemble model performing optimally using key features including preoperative radiotherapy, postoperative wound infection, plate exposure, and re-exploration within 30 postoperative days. SHapley Additive exPlanations (SHAP) analysis confirmed early postoperative complications as the primary predictors of ORN, supporting targeted monitoring of infection and plate-related issues and guiding clinical decisions regarding hyperbaric oxygen therapy, prophylactic antibiotics, and hardware removal to mitigate ORN risk. Kim et al. (27) constructed a Vision Transformer-based monitoring model for postoperative intraoral free flaps, which quantified vascular crisis probability using serial clinical images acquired every 2–3 h within 48 h postoperatively. In a representative case, the model detected a rise in risk probability from 1.3% to 13% within 3 h, despite clinicians rating the flap as unchanged; the patient subsequently required re-exploration. This work indicates that AI outperforms human visual assessment in detecting subtle flap alterations, offering a quantitative decision-support tool for early warning of postoperative vascular compromise.

### 5.2 Quantitative assessment of postoperative facial morphology

Facial asymmetry is one of the most common complications after CMF surgery and a leading cause of patient dissatisfaction (28). Traditional subjective assessment is highly influenced by physician experience and has poor reproducibility. A team from the University of Tokyo developed a free, lightweight, real-time AI facial asymmetry analysis software that automatically detects 68 facial landmarks using a standard laptop and quantifies displacement ratios of the mouth corners and brow peaks (29). The software automatically records time-series data, supports multi-timepoint comparison, and can be used to track surgical outcomes, identify complications early, and guide secondary repair timing. Postoperative craniofacial swelling, scar contraction, and tissue remodeling are highly individualized and difficult to predict using conventional methods. A team from Radboud University Medical Center developed a deep learning-based soft tissue prediction system for orthognathic surgery using 3D photography (30). The system repeatedly collects 3D facial images at multiple postoperative time points, automatically compares them with predicted morphology, generates full-face point-by-point error color maps, and objectively quantifies swelling resolution and soft tissue compliance changes, distinguishing between insufficient surgical effect and abnormal recovery. If deviations between actual and predicted soft tissues exceed 2 mm at 1 year postoperatively with patient dissatisfaction, the current facial morphology can be used to guide the timing and planning of secondary revision.

## 6 Discuss

### 6.1 Challenges of AI implementation in CMF surgery

#### 6.1.1 Data quality and model generalization dilemma

The AUROC value of the complication prediction model for cranioplasty established by Li et al. (7) was 0.949 in internal validation, which dropped to 0.930 in cross-regional external validation. The overall accuracy of the screening model for syndromic craniosynostosis developed by Hennocq et al. (13) was merely 70.2% on an independent international validation set, much lower than its performance on the training set. Such performance discrepancies indicate distribution shifts between single-center data and real-world data. The main causes of this shift are as follows: First, clinical data in CMF surgery are scattered across multiple systems, including CBCT, CT, MRI, and electronic medical records, with inconsistent annotation standards and large discrepancies in device parameters, making it difficult to construct high-quality training datasets (31). Second, variations in scan slice thickness and reconstruction algorithms among different brands of imaging equipment further aggravate the accuracy attenuation in cross-institutional applications (32). Third, most existing models are trained on single-center, standardized datasets, and their predictive and reconstructive performance drops substantially when dealing with complex defects, metal artifacts, and rare deformities in real clinical practice (33). A study by Li et al. (34) verified that even intensive data augmentation fails to cover all individualized skull defect morphologies.

#### 6.1.2 Methodological challenges of causal inference

Li et al. (7) identified the protective effects of negative pressure drainage and titanium mesh directly linking model outputs to intraoperative clinical decision-making. Matar et al. (26) used SHAP analysis to verify that postoperative infection and titanium plate exposure are critical warning indicators of ORN, and accordingly put forward targeted intervention recommendations. Identifying intervenable factors and guiding specific clinical decisions represents a pivotal leap for AI models to move from academic publication to clinical acceptance. However, the validity of causal inference is highly dependent on the control of unobserved confounding factors (35). Variables included in current studies are mostly restricted to easily accessible structured data from electronic medical records, such as age, gender, and laboratory indicators. Potential confounders including surgical team experience, quality of postoperative care, patient compliance, and socioeconomic status are not measured, which may undermine the accuracy and unbiasedness of ATE estimation.

#### 6.1.3 Model interpretability and clinical trust crisis

The black-box nature of AI models represents a major obstacle to clinical translation. The decision-making process of deep learning models lacks interpretability, making it difficult for clinicians to understand the rationale behind predictions and thus reducing trust and acceptance in clinical decisions (36). Compared with statistical shape models that explicitly represent shapes, latent features learned by deep neural networks are difficult to interpret, hindering clinical translation (34).

#### 6.1.4 Challenges in clinical translation

Outstanding challenges also exist in the clinical translation of artificial intelligence (AI) technologies into CMF surgery. Existing AI systems are mostly independently developed, closed platforms, and seamless integration with existing clinical workflows such as hospital information systems (HIS), picture arch and communication systems (PACS), and surgical navigation systems remains immature. The issue of data islands prevents AI models from acquiring real-time, complete, high-quality multidimensional data in real clinical settings (37). Meanwhile, the high costs of infrastructure including surgical robots, three-dimensional photography equipment, and high-performance computing hardware further limit the popularization of AI technologies in primary medical institutions (1).

### 6.2 Future prospects

Despite the challenges, the future of AI in craniofacial surgery is promising. Addressing these challenges requires a concerted effort across multiple fronts.

First, improving data quality and model generalizability. The performance degradation observed from internal to external validation underscores the urgent need for standardized, multi-center databases. Future efforts should establish a large-scale, multi-institutional registry for perioperative management in CMF surgery, with unified data annotation standards and harmonized imaging protocols. Data sharing and federated learning approaches may also enable model training across institutions without compromising patient privacy.

Second, advancing causal inference and actionable decision support. Moving beyond pure prediction, future AI models should integrate causal inference frameworks to identify modifiable risk factors and guide specific clinical interventions. Methods such as double machine learning, targeted maximum likelihood estimation, and Bayesian causal forests hold promise for estimating treatment effects from observational data, thereby supporting evidence-based surgical decision-making.

Third, enhancing model interpretability and clinical trust. The black-box nature of deep learning remains a major barrier to clinical adoption. Yang et al. (38) developed an automated skeletal malocclusion classification model that uses gradient-weighted class activation mapping (Grad-CAM) to generate heatmaps visualizing the facial regions on which the model focuses. This transparent decision-making process allows clinicians to intuitively verify the rationality of model judgments and enhance trust in AI. Future research should prioritize explainable AI (XAI) techniques, including attention visualization, SHAP analysis, and counterfactual explanations. These tools can help clinicians understand the rationale behind AI predictions, fostering trust and facilitating human-AI collaboration. Regulatory frameworks for AI validation and continuous monitoring should also be established.

Fourth, accelerating clinical translation and real-world implementation. Seamless integration of AI systems into existing clinical workflows—including hospital information systems (HIS), picture archiving and communication systems (PACS), and surgical navigation platforms—is essential. Future work should focus on developing standardized application programming interfaces (APIs), reducing hardware costs, and conducting prospective clinical trials to validate AI tools in real-world settings. Partnerships between academic institutions, industry, and regulatory bodies will be critical for successful translation.

Fifth, leveraging emerging AI technologies. Generative AI, including large language models (LLMs) and diffusion models, opens new avenues for personalized implant design, postoperative outcome visualization, and doctor-patient communication. Multimodal AI, integrating 3D imaging, genomics, electronic health records, and patient-reported outcomes, promises to deliver truly individualized perioperative management.

In summary, the path forward requires a multidisciplinary, stepwise approach: from building high-quality databases and developing explainable algorithms to conducting prospective validation and achieving routine clinical deployment. With continued progress in these directions, AI has the potential to transform perioperative care in craniomaxillofacial surgery, shifting from experience-driven to data-driven, personalized management.

## 7 Conclusion

AI has been comprehensively integrated into the entire perioperative workflow of CMF surgery, showing significant advantages in preoperative risk warning, personalized repair planning, bleeding and transfusion prediction, intraoperative intelligent navigation, autonomous robot-assisted surgery, postoperative complication risk stratification, and objective assessment of morphological and soft tissue recovery. These advances effectively promote the shift from experience-driven to data-driven diagnosis and treatment. Current research still faces challenges including non-uniform data standards, insufficient model interpretability, and slow clinical translation. With continued progress in multimodal data fusion, explainable AI, and generative AI, artificial intelligence will further support the construction of full-cycle, personalized intelligent management systems covering preoperative, intraoperative, and postoperative phases, continuously improving the safety and efficacy of CMF surgery and facilitating the implementation of precision medicine.
