# Supplementary material for: AI-driven perioperative risk stratification and complication management in craniomaxillofacial surgery: current progress and future directions
Source: Front Surg. 2026 Jul 6;13:1859732. doi: 10.3389/fsurg.2026.1859732 (PMC13381262; doi:10.3389/fsurg.2026.1859732)
Supplement: Supplementary file 1 [file Table1.docx]

Supplementary Material

# Supplementary Table S1. Characteristics and Clinical Translation Status of AI Studies in Craniomaxillofacial Surgery

| Section | Citation | Study Design | Validation Type | Sample Size | AI Method | Performance Metrics | Clinical Translation Status |
| --- | --- | --- | --- | --- | --- | --- | --- |
| 2.1 Intelligent Early Recognition of Syndromic Craniosynostosis | Hennocq et al. (12) | Diagnostic study | Independent international external validation | 541 patients, 2,228 images | Deep learning + XGBoost classifier | Overall accuracy 70.2%; Crouzon-Pfeiffer group balanced accuracy 84.4%, AUC 0.941 | Research stage (AI screening tool) |
|  | Hennocq et al. (13) | Case report | Real-world image test | Trained on the same database | AIDY algorithm (based on the above model) | Successful identification of Apert syndrome from low-resolution news photograph | Research stage (humanitarian application) |
| 2.2 Personalized Cranial Repair and Intelligent Implant Design | Juneja et al. (SCAI-Net) (6) | Methodological study | Internal validation | 2,760 synthetic skull defects | Deep learning (V-Net architecture) | DDR: DSC 0.889, HD 1.856 mm; design time 93.5 s (CAD: 2,820 s) | Research stage (methodology development) |
|  | Friedrich et al. (15) | Methodological study | Internal validation | — | Point cloud diffusion model | Implant generation in ~20 min, provides multiple candidate options | Research stage (methodology development) |
|  | Zubizarreta-Oteiza et al. (16) | Methodological study | Internal validation | 3 molds | Generative design algorithm | Design time: 2 h → 1.3 min; accuracy: 0.0763 mm | Research stage (preclinical validation) |
| 2.3 Complication Risk Prediction | Stehrer et al. (18) | Prediction study | Internal validation | 950 patients | Random forest | Strong correlation with actual blood loss (p<0.001), mean deviation 7.4 ml (SD 172.3 ml) | Research stage |
|  | Li et al. (7) | Prediction/causal inference study | Internal + geographic external validation | 1,368 patients (multicenter) | Random forest + DML + T-learner + SHAP + DiCE | Overall complications: AUROC 0.949 (internal), 0.930 (geographic), 0.932 (temporal); Seven specific complications: AUROC 0.851–0.986; Causal effects: drainage ATE=-0.241, titanium mesh ATE=-0.191 | Research-to-clinical translation (public web tool developed) |
| 3.1 AI-Driven Augmented and Mixed Reality Navigation | Xie et al. (20) | Methodological/technical validation study | Laboratory validation (phantom + cadaver experiments) | — | SVD algorithm + Kalman filtering | Mean tracking error 1.55±0.25 mm, system delay 75–100 ms | Research stage (preclinical validation) |
|  | Medtronic Stealth AXiS (21) | Product report | FDA approved | — | Intelligent planning + optical navigation + robotics | — | Commercialized (FDA approved March 2026) |
| 3.2 Autonomous Planning Surgical Robots | Lin et al. (22) | Preliminary clinical study | Internal validation | 6 patients | Adaptive fuzzy control + Kalman filtering | Automatic bone drilling control, autonomous stop based on force perception | Research stage (preliminary clinical application) |
|  | Guo et al. (23) | Case report | Internal validation | 1 patient | Autonomous dental implant robot (intelligent planning + visual tracking + force servo) | Precise implantation of multiple implants and immediate restoration under limited bone volume | Clinical application |
|  | Li et al. (24) | In vitro comparative study | Internal validation (in vitro model experiment) | 60 implants (in vitro) | Autonomous planning + human-robot collaborative hybrid model | Hybrid technique: slightly lower accuracy (~1 mm deviation, angle <5°); time reduced by 66% (206 s vs 605 s, P<0.001) | Preclinical (in vitro) |
| 4.1 Early Warning of Postoperative Complications | Matar et al. (25) | Retrospective cohort study (prognostic prediction) | Internal validation (single-center retrospective data) | 275 oral cancer patients receiving radiotherapy | 5 ML algorithms: Stacked Ensemble, DNN, KNN, LightGBM, SVC | Confirmed early complications (infection, plate exposure) as primary predictors | Research stage (toward clinical translation) |
|  | Kim et al. (26) | Retrospective diagnostic/monitoring model study | Internal validation | 131 patients, 1,877 images | Vision Transformer | Overall accuracy 0.9867, F1 score 0.9863; minority class precision 0.95/recall 0.83; 5-fold patient-level CV accuracy 0.938 | Research stage |
| 4.2 Quantitative Assessment of Postoperative Facial Morphology | Takeaki et al. (28) | Methodological study | Technical demonstration / proof-of-concept | — | Incremental parallel cascade of linear regression algorithm for automatic detection of 68 facial landmarks | Real-time quantification of oral commissure and eyebrow vertex displacement ratios | Research stage (free software) |
|  | Berends et al. (29) | Retrospective model development and validation study | Internal validation | 458 orthognathic surgery patients | Morphable model + PCA + feedforward neural network | Mean prediction accuracy 1.17±0.49 mm; prediction speed <0.02 s | Research stage |

Note: AUC, area under the curve; AUROC, area under the receiver operating characteristic curve; ATE, average treatment effect; CAD, computer-aided design; CBCT, cone beam computed tomography; CV, cross-validation; DDR, direct defect reconstruction; DiCE, diverse counterfactual explanations; DML, double machine learning; DSC, Dice similarity coefficient; FDA, Food and Drug Administration; HD, Hausdorff distance; ML, machine learning; PCA, principal component analysis; SD, standard deviation; SHAP, SHapley Additive exPlanations; SVD, singular value decomposition; XGBoost, extreme gradient boosting. "—" indicates not reported or not applicable. "Research stage" indicates internal validation completed but not yet routinely applied in clinical practice; "Clinical application" indicates preliminary application in real patients; "Commercialized" indicates approved product available on the market; "Preclinical" indicates validation completed in vitro or in animal models.
